# Recent Emergence of Rhenium(I) Tricarbonyl Complexes as Photosensitisers for Cancer Therapy

**DOI:** 10.3390/molecules25184176

**Published:** 2020-09-12

**Authors:** Hui Shan Liew, Chun-Wai Mai, Mohd Zulkefeli, Thiagarajan Madheswaran, Lik Voon Kiew, Nicolas Delsuc, May Lee Low

**Affiliations:** 1 School of Postgraduate Studies, International Medical University, Bukit Jalil, Kuala Lumpur 57000, Malaysia; liew.huishan@student.imu.edu.my; 2 Centre for Cancer and Stem Cell Research, International Medical University, Bukit Jalil, Kuala Lumpur 57000, Malaysia; chunwai_mai@imu.edu.my; 3 School of Pharmacy, International Medical University, Bukit Jalil, Kuala Lumpur 57000, Malaysia; mohdzulkefeli@imu.edu.my (M.Z.); thiagarajan@imu.edu.my (T.M.); 4 Department of Pharmacology, Faculty of Medicine, University of Malaya, Kuala Lumpur 50603, Malaysia; lvkiew@um.edu.my; 5 Laboratoire des Biomolécules, Département de Chimie, École Normale Supérieure, PSL University, Sorbonne Université, 75005 Paris, France; nicolas.delsuc@ens.psl.eu

**Keywords:** cancer, medicinal inorganic chemistry, metals in medicine, photodynamic therapy, photosensitisers, rhenium(I) tricarbonyl complexes

## Abstract

Photodynamic therapy (PDT) is emerging as a significant complementary or alternative approach for cancer treatment. PDT drugs act as photosensitisers, which upon using appropriate wavelength light and in the presence of molecular oxygen, can lead to cell death. Herein, we reviewed the general characteristics of the different generation of photosensitisers. We also outlined the emergence of rhenium (Re) and more specifically, Re(I) tricarbonyl complexes as a new generation of metal-based photosensitisers for photodynamic therapy that are of great interest in multidisciplinary research. The photophysical properties and structures of Re(I) complexes discussed in this review are summarised to determine basic features and similarities among the structures that are important for their phototoxic activity and future investigations. We further examined the in vitro and in vivo efficacies of the Re(I) complexes that have been synthesised for anticancer purposes. We also discussed Re(I) complexes in conjunction with the advancement of two-photon PDT, drug combination study, nanomedicine, and photothermal therapy to overcome the limitation of such complexes, which generally absorb short wavelengths.

## 1. Introduction

Cancer has caused approximately 10 million deaths around the world [1]. Despite the advancement in cancer diagnosis and therapy, it is still the second leading cause of death even in the United States [2]. Conventional cancer therapies include surgery, radiotherapy, and chemotherapy [3,4]. Surgery is the mainstay of treatment for most of the solid tumours. Unfortunately, not all tumours are resectable [5,6]. Cancer patients who can undergo radiotherapy would have better long-term survival, tumour cure, and local tumour regression. However, radiotherapy may induce metastatic spread, tissue complications, and high rates of loco-regional failure [7,8,9,10]. For patients who cannot receive surgery or radiotherapy, chemotherapy has been the mainstay of treatment. Cisplatin, gemcitabine, and doxorubicin are some of the commonly prescribed chemotherapies for most cancer patients. However, their poor cellular penetration and adverse side effects may limit their therapeutic efficacy [11]. Although the recently emerging immunotherapy has promising therapeutic efficacy in selected solid tumours, its efficacy is often unpredictable due to the variability in the host’s immune system and the complicated cancer-immune crosstalk [12,13]. The cancerous cells might develop resistance towards both chemotherapy and immunotherapy when its efficacy drops to the sub-therapeutic zone. Hence, there is an urgency to develop more alternative approaches to prevent and to overcome drug resistance [14]. One of such alternatives would be to utilise photodynamic therapy (PDT).

**Figure 1:**
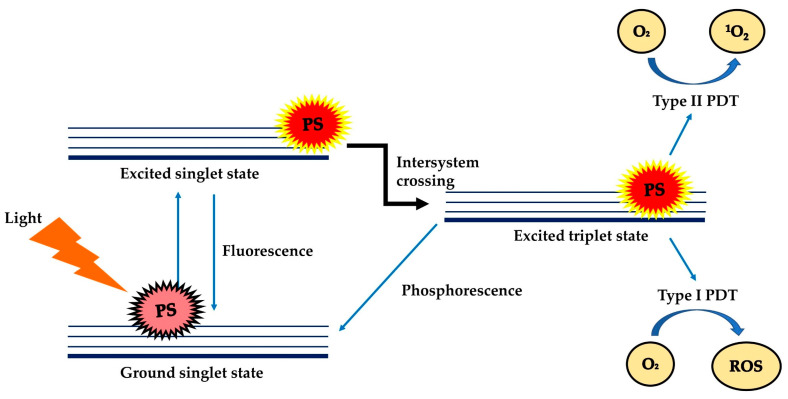
Schematic illustration of the mechanism of PDT which involves Type I and Type II mechanisms. PS: photosensitiser; O_2_: oxygen; ^1^O_2_: singlet oxygen; ROS: reactive oxygen species.

PDT is a treatment modality that uses harmless visible light to activate non-toxic light-excitable molecules (i.e., the photosensitisers), for the generation of cytotoxic reactive oxygen species (ROS, type I photoactivation) and singlet oxygen (type II photoactivation) from molecular oxygen (Figure 1) [15,16,17,18,19,20,21] within or at near vicinity to cancer cells, to cause cell death and tissue necrosis [22]. PDT was claimed to be relatively selective and safe compared to classical anticancer therapies due to the use of harmless therapeutic agents during the process, liberty of activating only the photosensitisers (PSs) at the tumour region via selective exposure of the tumour to light, and increased tendency of the targeted photosensitisers to accumulate at the tumour site [21,23]. PDT can be used repetitively without causing resistance to tumour or hypersensitivity to normal tissue, as a single agent or in conjunction with other anticancer therapies such as chemotherapy and radiotherapy [24,25,26,27,28].

At present, a number of PSs have received approval from the FDA for the treatment of cancer (e.g., skin, pancreatic and other cancers—refer to Table 1) [22] and non-cancer disorders (e.g., verteporfin for age-related macular degeneration treatment and 5-aminolevulinic acid for moderate to severe acne vulgaris) [29]. Despite the advantages, the extensive clinical use of PDT was limited by the inherent weaknesses of common photosensitisers, such as water insolubility, aggregation tendency in the physiological environment, and requirement of a rich oxygen environment for the production of singlet oxygen/ROS [30,31,32,33,34]. In recent years, Re(I) complexes have been increasingly explored as a new alternative choice of photosensitisers for PDT [19,35,36]. This review gives a brief account of the development of anticancer PDT and assesses the potential of Re(I) complexes as potent photosensitisers for PDT.

## 2. Photosensitisers for Photodynamic Therapy—A Brief History

Photosensitisers (PS) were first introduced as an anticancer treatment on a commercial scale by Thomas Dougherty and his co-workers in the 1970s. Since this pioneering work, three generations of photosensitisers have been developed [48,54].

### 2.1. First and Second Generation Photosensitisers

**Figure 2:**
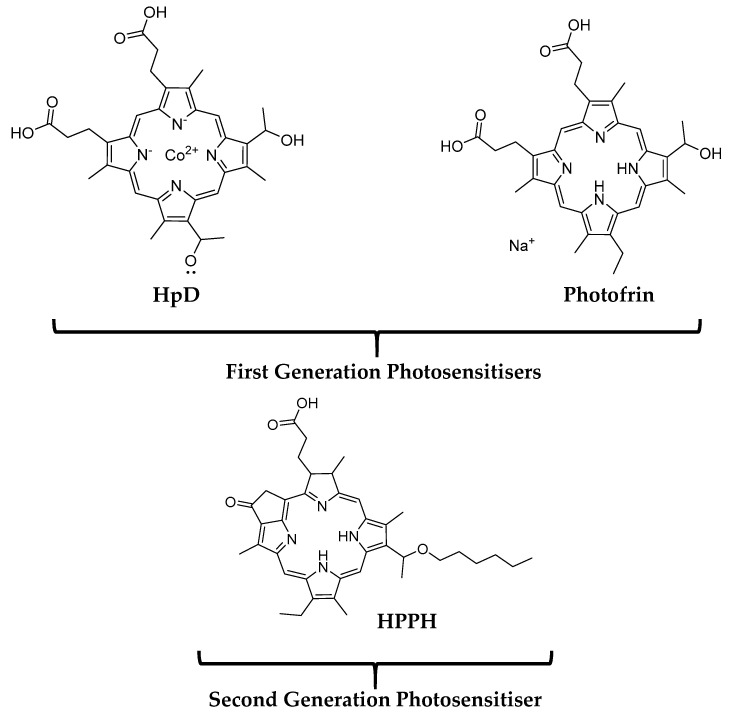
Structures of a few examples of common photosensitisers.

The first generation of PDT PSs was mainly porphyrin-based, notable examples include hematoporphyrin derivatives (HpD) and Photofrin (porfimer sodium) for the treatment of skin cancer (shown in Figure 2) [55]. The use of first-generation PSs for anticancer purposes was not well received due to their poor tissue penetration, low chemical purity and long half-life, which results in high accumulation in the skin thus causing skin hypersensitivity [56]. In an attempt to address these weaknesses, the development of second-generation photosensitisers was initiated in the early 1980s [57,58,59]. The examples of second-generation photosensitisers include chlorin, benzoporphyrin derivatives and hematoporphyrin derivatives [60]. Compared to their predecessors, second-generation photosensitisers can be synthesised at a higher purity, absorb light that better penetrates the body tissues (range of wavelength: 650–800 nm), produce a higher amount of singlet oxygen and cause fewer side effects. For instance, highly lipophilic 2-[1-hexyloxyethyl]-2-devinyl pyropheophorbide-a (HPPH), as known as Photochlor (shown in Figure 2), has been evaluated extensively and approved in the USA to treat head, lung and neck cancers. Photochlor demonstrates enhanced pharmacokinetic properties and only result in mild skin photosensitivity which undergoes a rapid reduction after a few days of administration of Photochlor in comparison with the first generation of PSs [44,45,46]. Nevertheless, the second generation photosensitisers are still poorly water-soluble. This limits their intravenous (IV) use in clinical settings [32].

### 2.2. Third Generation Photosensitisers

The third generation of PSs was developed to enhance their bioavailability to the targeted tissue [61]. They were either novel compounds that possess higher selectivity and affinity to cells of the targeted tissue (e.g., cancer) than normal tissues or nano-complexes formed by combining selected second-generation photosensitisers with suitable antibodies [62,63] or active/passive disease-tissue-targeting nano-drug-carriers, such as peptides, polymers and polymeric nanoparticles. One example of such nano-carrier-photosensitiser complexes was chlorin E6 (Ce6) complexes incorporated into ursodeoxycholic acid-conjugated chitosan nanoparticles that showed a promising anticancer effect towards HuCC-T1 (human cholangiocarcinoma cells) [64]. Similarly, conjugation of protein (Shiga-like toxin B subunit) to Ce6 to specifically target the overexpressed receptors in ovarian cancers, glycosphingolipid receptor Gb_3_ has caused an improved photodynamic anticancer effect on the in vitro ovarian cancer cells by a factor of 10 in comparison with Ce6 alone [65,66]. Of particular interest in the second and third generation of PDT photosensitisers is the use of metal-based drugs, especially ruthenium [67,68,69,70,71,72]. As a significant highlight, the ruthenium polypyridyl complex TLD1433 of the McFarland group is currently in phase II clinical trials against bladder cancer [73,74].

### 2.3. Characteristics of an Ideal Class of Photosensitisers for PDT

Considering the numerous stringent requirements that need to be met in pharmaceutical production and clinical use, an ideal photosensitiser should possess the following characteristics (i) able to generate singlet oxygen even in a hypoxia state and high quantum yield of ^1^O_2_, (ii) chemically pure and stable at room temperature, (iii) able to be synthesised through an easy, straightforward, and inexpensive method which allows consistent and reproducible product, (iv) possess high photochemical activity, molar absorption coefficient and absorbs tissue penetrating light (around 650 nm to 800 nm) for the production of reactive oxygen species, (v) possess good photochemical reactivity with long lifetimes of triplet state and high triplet state yields (for the efficient production of singlet oxygen or reactive oxygen species upon irradiation), (vi) the absorption band of the PS must not overlap with the absorption band of the substances located in the body such as hemoglobin, melatonin, (vii) selectively phototoxic at a defined wavelength, (viii) solubilise easily in the body tissues to keep appropriate lipophilic ability to cross phospholipid cell membrane and avoid tendency to self-aggregate in biological environment, (ix) can be easily cleared from normal tissue and do not cause dark toxicity, (x) selectively accumulate at the targeted tissue and (xi) be a pain-free and reliable PDT treatment [28,75,76,77,78,79]. With reference to the above, Re(I) tricarbonyl complexes meet most of the requirements as metal-based phosphorescent PSs except for the excitation wavelength in which this class of compounds generally absorbs short wavelengths below 400 nm.

## 3. Rhenium(I) Tricarbonyl Complexes: General Overview

### 3.1. Background of Rhenium (Re)

Re, a neighbour of Ru in the periodic table is a rare element in the earth. It possesses a mean concentration of around 0.5 to 1 ppb in the earth’s crust, which is lesser than the other third-row transition metals [80,81,82]. Generally, Re occurs in little amounts in minerals and ores but not in its elemental form in nature. Re exists as two natural isotopes, ^185^Re (37.4%) and ^187^Re (62.6%). Both isotopes are potential candidates for medical applications. ^187^Re is a beta emitter that possesses a long-decay half-life but is proposed to be safe for medical use owing to its relatively weak emission of radiation [83,84,85,86,87,88]. It has been evaluated as an anticancer drug candidate in clinical trials previously in the form of organic-metal complexes [89,90,91,92,93].

### 3.2. Re(I) Complexes: Brief History and Suitability as a PDT PS

**Figure 3:**
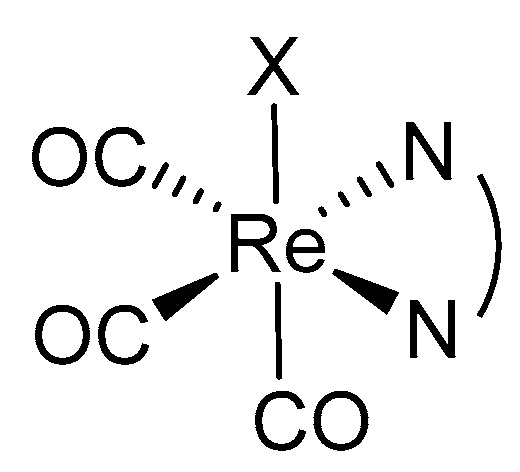
General structure of Re(I) tricarbonyl core with variable X depending on the ligands incorporated to form different Re(I) tricarbonyl complexes with different phototoxic activity on cancer cell lines.

The synthesis of Re(I) carbonyl Re(I)(CO)_3_^+^ complexes was first performed by Walter and co-workers in 1941. In general, the core of Re(I)(CO)_3_^+^ complexes (shown in Figure 3) is hard Lewis acid-based and stable in aqueous solutions, even in dilute hydrochloric acid and coordinating solvents [94,95]. Re(I) tricarbonyl complexes possess more photophysical and chemical features that meet the requirements of ideal photosensitisers when compared to other metal complexes and some conventional PSs. For instance, Re(I) tricarbonyl complexes exhibited polarised emission, large Stokes shifts, high photostability, and long lifetimes [96,97,98]. They are also more biocompatible due to the presence of a low spin d^6^ electronic configuration at the Re(I)’s outermost shell. Such configuration makes the Re(I) tricarbonyl complexes kinetically inert, and thus devoid heavy metal related toxicity [99]. However, in terms of photoreactivity, native Re(I) tricarbonyl complexes were activated by light of short wavelength (350 nm) to generate ROS [100]. This naturally limits their use as PDT PSs due to the low tissue penetration characteristics of short-wavelength light. Nevertheless, ongoing efforts of structural modifications and the recent advancement using two-photon PDT technology, photothermal therapy, drug combination study, as well as the incorporation of rhenium(I) complex into nanoparticles are potential strategies to overcome this main drawback.

## 4. Phototoxic Effect of Rhenium(I) Tricarbonyl Complexes

### 4.1. Rhenium(I) Tricarbonyl Complexes in PDT: Modifications and Advancements

Despite being natively activated by low-tissue-penetrating lights, the relatively reduced heavy metal toxicity and interesting photophysical and photochemical properties of Re(CO)_3_ complexes when compared to other metal-based PSs have attracted ongoing interests and efforts to improve this class of compounds for PDT [101]. The attempted approaches were described as follows.

### 4.2. Structural Modification for the Enhancement of Re(I) Complexes’ Phototoxicity, Tissue Selectivity and Photo-Stability

**Figure 4:**
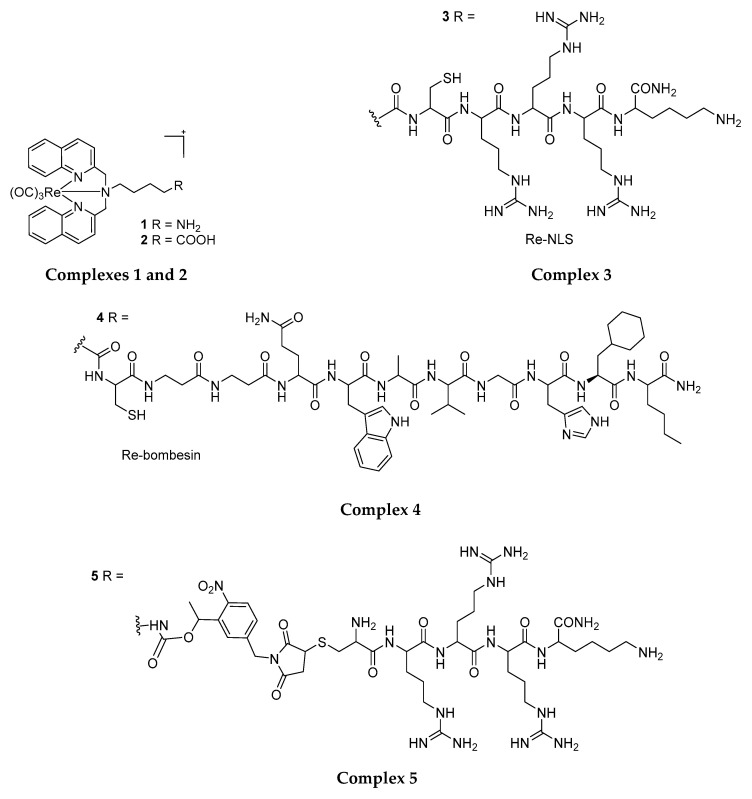

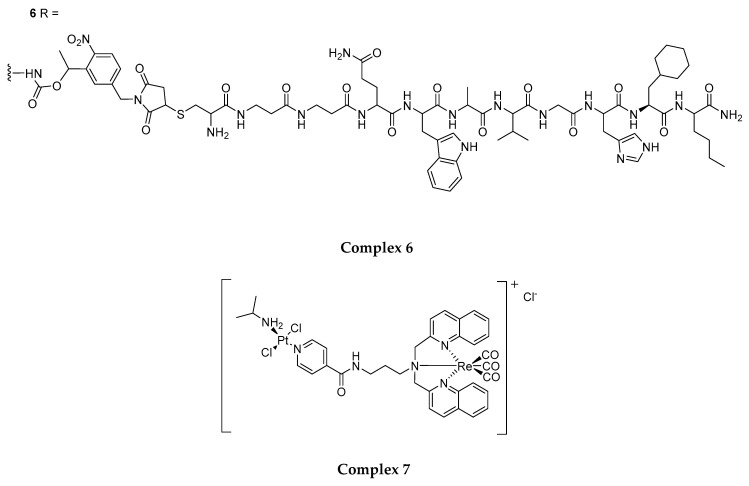
A series of different ligands complexed to the Re(I) tricarbonyl core forming different Re(I) tricarbonyl complexes with different phototoxic activity on cancer cell lines.

Gasser et al. (2014) attempted to enhance the phototoxicity, tissue selectivity and photo-stability of Re(CO)_3_ complexes through structural modification and targeting ligands/protecting groups conjugation. The first products of the group, i.e., amino and carboxylate functionalised N,N-bisquinoline Re(I) tricarbonyl complexes (**1** and **2** in Figure 4) [35] showed phototoxicity toward HeLa cells (IC_50_: 17.3 µM and 9.3 µM respectively) and no dark toxicity (IC_50_: > 100 µM) upon UVA light irradiation at 350 nm (fluence rate of 2.58 J cm^−2^). Conjugation of **1** and **2** respectively with an NLS peptide (nuclear localisation signal, to yield **3**) to target the nucleus and bring the complex in close proximity of DNA and bombesin (to yield **4**), a neuropeptide has known to selectively target cancer cells over healthy cells further improved the phototoxicity of these compounds against HeLa cells (IC_50_: 18.3 µM and 5.3 µM respectively). This exhibits the possibility and flexibility to improve the cell selectivity and phototoxic effects of these PSs using cell-targeting ligands [35]. Gasser et al. further explored the use of a photolabile protecting group (PLPG) to enable the release of **1** at low irradiation energy (1.2 J cm^−2^). Such a strategy combining targeting with peptides and irradiation-dependent release of the Re complex (yield **5** and **6**) has again increased the phototoxicity of these compounds against cancer cells (HeLa and PC-3, IC_50_: 9.3 µM and 9.7 µM respectively) while, at the same time, reduced the phototoxic effects towards non-cancerous cells (MRC-5)(20.5 µM and 23.3 µM respectively) [19].

Meanwhile, Quental et al. (2017) designed a heterobimetallic complex consisting of a Re(I) tricarbonyl core and a platinum-iminedipyridyl group. Addition of platinum into the Re(I) complex (complex **7**) was found to be capable of improving the toxicity of Re(I) by three folds towards HeLa cell lines (IC_50_ from 42.8 µM to 13.5 µM, upon 10 min light irradiation at 350 nm and 2.58 J cm^−2^), achieving a similar photo-index value (PI) to that of the Photofrin (PI: average of 5) [35,102,103]. This suggests the potential for improving Re(I)’s phototoxicity via attachment of a Pt(II) cytotoxic complex.

### 4.3. Structural Modification for the Improvement of Re(I) Complexes’ Photo-Absorption Profiles

The phototoxicity and PDT compatibility of the Re(I) complexes can be enhanced through improving the light absorption profiles of these compounds.

**Figure 5:**
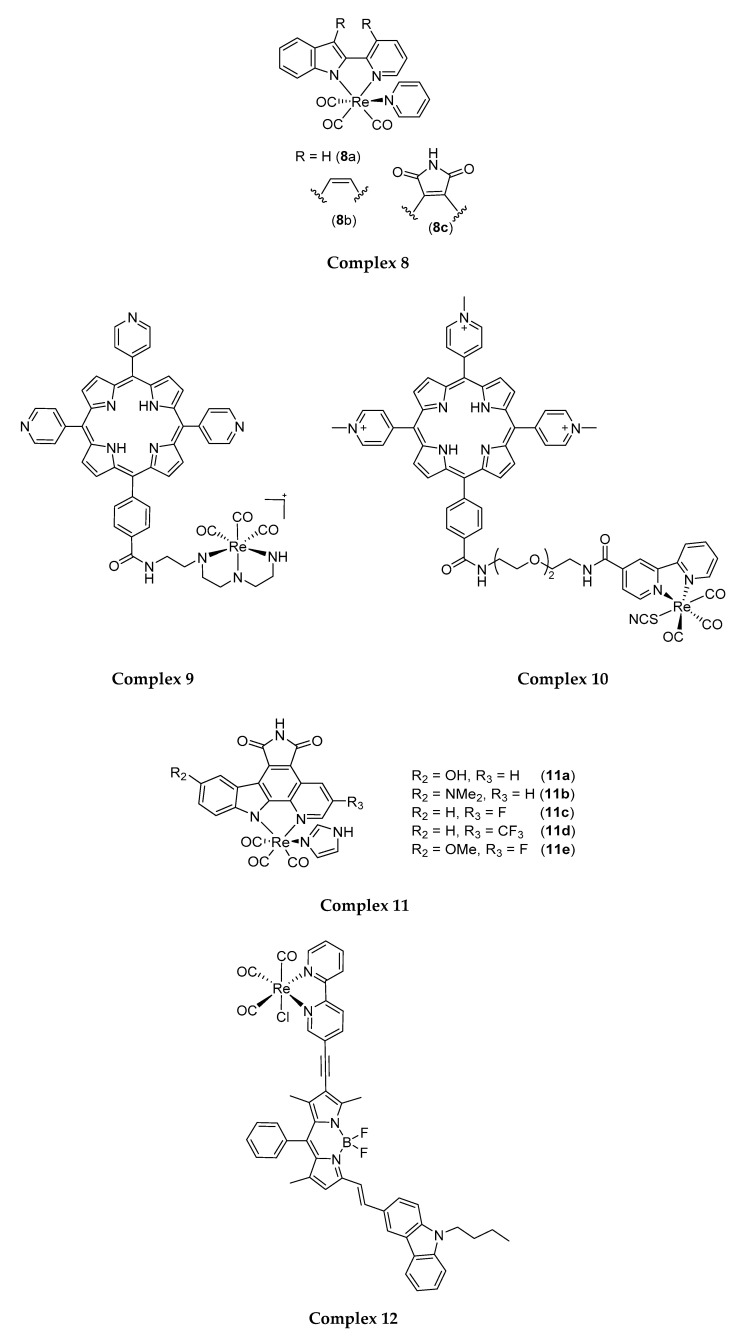

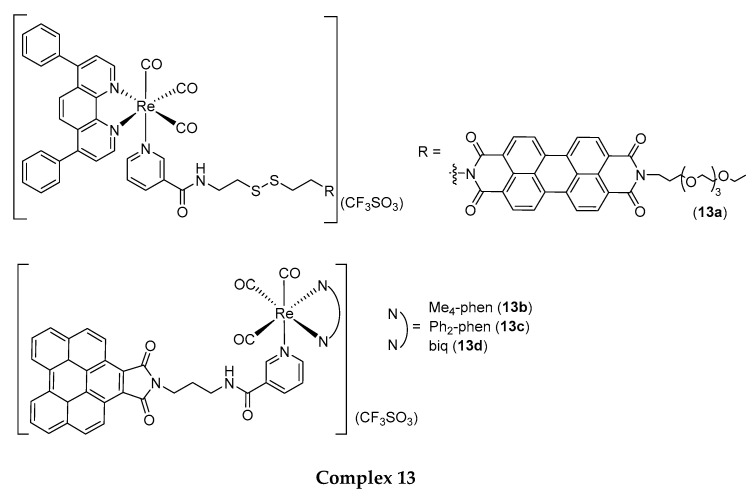
A number of different ligands complexed to the Re(I) tricarbonyl core forming different Re(I) tricarbonyl complexes with different photo-absorption profiles.

Following this approach, Meggers and co-workers (2013) synthesised Re(I) tricarbonyl indolato complexes (**8** in Figure 5) that can be activated by light of λ > 505 nm. The most potent derivative **8c** was found to be capable of inducing phototoxicity on HeLa cells (IC_50_: 0.1 µM) and was localised at the edges of melanoma spheroids to induce loss of spheroid integrity and decrease spheroid size [104,105]. Interestingly, the addition of a pentene ring with nitrogen and oxygen substitution to complex **8c** made it more lipophilic, easier to penetrate through cell membranes, and thus result in better phototoxic activity towards HeLa cells.

Gianferraraand co-workers (2014) synthesised water-soluble porphyrin-Re conjugates bearing with a diethylenetriamine **9** or a bipyridine ligand **10** that can be excited at 590–700 nm (fluence rate of 9 mW cm^−2^). These porphyrin-Re conjugates **9** and **10** were found to be phototoxic against HeLa cancer cells (IC_50_: 1.9 µM and 4.0 µM, respectively). Nevertheless, the phototoxic activity of these complexes was later attributed to the porphyrin moiety, as the Re complex does not produce any singlet oxygen upon 590–700 nm irradiation [106].

In another attempt, Ludewig et al. (2014) produced Re(I) pyridocarbazole complexes that induce cancer cell apoptosis post-irradiation with light of long visible wavelengths (600–850 nm) [104]. Modification of pyridocarbazole heterocycle with different functional groups was also attempted to achieve redshifts in the light absorption profile of the PSs (**11a**–**11e**). This includes the introduction of a dimethylamino group and π-donating hydroxyl group onto the indole moiety at the 5th position; trifluoromethyl and σ-accepting fluoride group onto the pyridine at the 3rd position; and π-donating methoxyl group to the indole combine with a σ-accepting fluoride in pyridine moiety [107]. Among these new complexes, **11c** and **11d** generated singlet oxygen at λ ≥ 620 nm and showed enhanced phototoxicity upon irradiation for 60 min with 7W LED light. Complex **11c** with fluorine at the 3rd position of the pyridine moiety showed the most significant red light-induced phototoxic effect on HeLa cells with an EC_50_ of 0.3 µM [108]. The current study suggested the feasibility of performing structural modification using Re(I) tricarbonyl-pyridocarbazole complexes as a scaffold to improve Re(I) complexes’ photo-absorption profiles, and pointed a potential direction for the development of tissue-penetrating near-infrared light-absorbing Re(I) complexes, for effective clinical PDT.

Zhong and co-workers (2015) published another Re(I) tricarbonyl complex linked with bipyridine (bpy) and boron dipyrromethene (bodipy). Bpy is a polypyridyl ligand while bodipy is a visible light-harvesting chromophore whose molecular structure can be readily derivatised [109,110,111,112]. Attachment of bodipy to the Re(I) tricarbonyl core (compound **12**) increased the λ_max_ of the Re(I) complexes from 399 nm to around 635 nm, and at the same time, confers to the Re(I) complexes the ability to produce long-lived triplet state (life-time of 448.9 µs) compared to that of the Re(I) complex conjugated only to bipyridine (2.22 µs) [113]. Such characteristics are important for the efficient production of singlet oxygen in an environment of low dissolved oxygen concentration such as cancer interstitium. Complex **12** was subsequently tested on LCC (lung cancer cells) upon irradiation at 635 nm using an LED light irradiation for 4 h, but was found to only produce minimal phototoxic effects [112]. Although unable to exhibit good phototoxicity, it may be worthwhile to revisit the current design to identify the pitfalls and produce a functional molecule, in viewing at the feasibility of the concept.

Another recent study by Alex et al. (2019) reported that luminescent Re(I) tricarbonyl complexes bearing either a perylene diimide (complex **13a**) or benzoperylene monoamide moiety (BPMI) (complex **13b**, **13c**, **13d**) showed significant phototoxic effect on HeLa cells after 15 min of 6 W UV-A irradiation at 365 nm (IC_50_: **13a**: 18.21 µM; **13b**: 0.27 µM; **13c**: 2.21 µM; **13d**: 1.51 µM). Among the four Re(I) complexes, complex **13b** appeared to be the most efficacious with an IC_50_ of 0.27 µM. This has been attributed to its longer lifetime, which is likely caused by the presence of the Re(I) core, enhancing intersystem crossing to populate excited triplet state of BPMI [114]. Therefore, the Re(I) tricarbonyl core with phenanthroline (phen) ligand complexed to it is important for its luminescent long lifetime properties, thus causing a strong phototoxic effect.

## 5. Re(I) Tricarbonyl Complexes: In Vivo Studies

**Figure 6:**
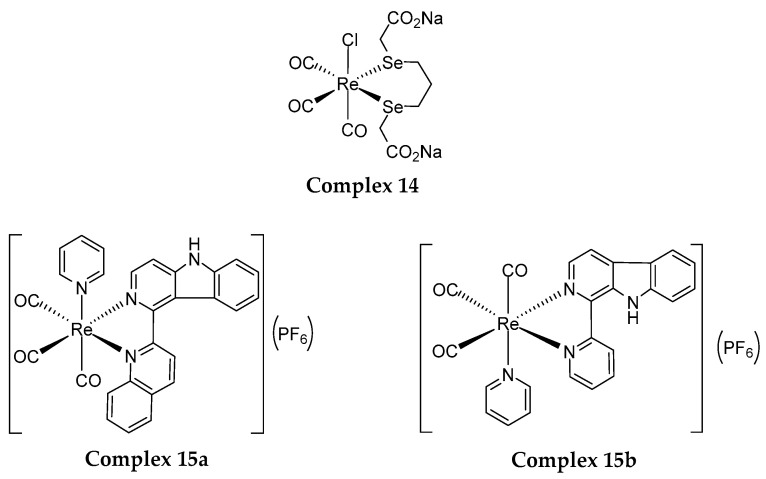
Different ligands complexed to a Re(I) tricarbonyl core forming different Re(I) tricarbonyl complexes with different cytotoxic activity on cancer cell lines.

Despite reports on Re(I) tricarbonyl complexes tested in vitro, there were only a few Re(I) complexes that went through in vivo testing. Collery et al. (2015) first reported a water-soluble, stable and lipophilic Re(I) tricarbonyl complex, which coordinates to two selenium (Se) atoms (Complex **14** as shown in Figure 6). Complex **14** showed good cytotoxic effect towards breast cancer cells and mice bearing with MDA-MB-231 Luc+ human breast cancer cells. Complex **14** was previously reported with an anticancer effect (IC_50_: 10 µM) towards MDA-MB-23l cells [115] and (IC_50_: 4.75 µM) MCF-7 breast cancer cells [116] before it proceeded to in vivo testing. Complex **14**, orally administered was then tested on mice that were transplanted with breast cancer cells as mentioned and it was found that a 10 mg/kg dose was safe, non-toxic, tolerable, and able to lessen tumour growth for at least four weeks of treatment [117,118]. This concludes the addition of Se to the Re(I) tricarbonyl core can efficiently treat breast cancer by targeting the cancer cell and its microenvironment.

In a more recent study performed by He and co-workers (2019), stable Re(I) tricarbonyl complexes that bind with β-carboline and pyridine ligands, complexes **15a** and **15b**, were synthesised and shown to kill A549 (human lung cancer cells) and A549R (cisplatin-resistant human lung cancer cells). Higher anticancer activity was achieved by **15a**, which displayed an IC_50_ of around 2 µM towards both cell lines mentioned as compared to **15b** (IC_50_: 4 µM). Then, they proceeded with in vivo testing of the most cytotoxic complex, complex **15a** on mice which were transplanted with A549 cancer cells. It is reported that a 5 mg/kg dose of complex **15a** is required to cause lysosomal dysfunction and cell death through autophagy over a three-week treatment [119]. The coordination of pyridine and β-carboline ligands did enhanced the anticancer activity of the Re(I) complex.

However, to our best knowledge, there is still no in vivo testing of the Re(I) tricarbonyl complex through PDT. This could be due, among others, to a wavelength of absorption that is too short.

## 6. Future Perspective—How to Improve Anticancer Activity of Re(I) Tricarbonyl Complexes in PDT?

Although there have been unsuccessful attempts, examples illustrated in the current review have also pointed out the possibility of improving the various photophysical and photochemical properties of Re(I) complexes, to address its inherent weakness (activation by short-wavelength light) and to improve its phototoxicity, tissue selectivity and photo-stability. Coupled with its reduced heavy metal toxicity [101], Re(CO)_3_ complexes may be an attractive candidate to be further investigated upon, for the generation of new effective PSs for PDT. The introduction of more ring structures or fluorine or methyl groups as displayed by reported complexes will be the structural strategy for improved lipophilicity and phototoxic effect of new Re(I) tricarbonyl complexes. Moreover, Re(I) complexes can be included in formulations with various nanoparticles such as liposomes, liquid crystalline nanoparticles or micelles to enhance its solubility and delivery to the cancer cells or be paired with synergistic agents like current chemotherapeutic agents to improve cellular targeting effect and further enhance treatment outcomes. Advancements in combination therapies discovery may unlock a new platform to strengthen the antitumor effects and enhance clinical outcomes. From our review, only a few Re(I) complexes advanced into the in vivo or clinical phase [120,121,122]. The in vivo studies mainly focused on Re(I) tricarbonyl complexes tested on the cytotoxic activity but not on PDT. This might be due to the short UV wavelength produced. With the emergence of two-photon laser technology, Re(I) complex can be further investigated to produce a deeper tissue penetration through a two times higher wavelength. Besides, the Re(I) complex may also be studied further in PTT to improve its anticancer activity through heat energy. As Re(I) complexes have been most frequently studied in cell culture, more preclinical and clinical studies are expected to be carried out in the future.

### 6.1. Two-Photon Photodynamic Therapy

**Figure 7:**
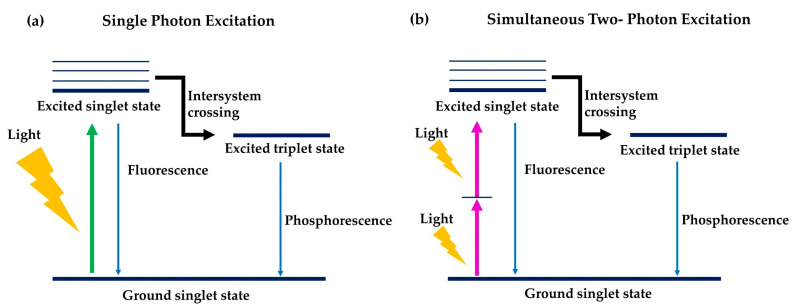
Comparison mechanism of PDT of single-photon excitation (**a**) and simultaneous two-photon excitation (**b**).

Two-photon absorption (TPA) is a nonlinear optical process that can be used to supply light energy to short wavelength-light-absorbing PSs for their photoactivation. In this process, two photons of lower energy i.e., at a longer wavelength (2× that of the short-wavelength photon normally absorbed by the PS) are supplied simultaneously to a short wavelength-light-absorbing PSs. These photons will be absorbed by the PSs and their energy combined to promote the PSs to an excited state. Such combined energy would be similar to the energy supplied by one short wavelength-photon for excitation of PSs (Figure 7). One important factor that needs to be considered in TPA is the ability of PS to absorb two photons concurrently, which is quantified by the TPA cross-section (δ), expressed as Goeppert–Mayer units (GM) [123,124,125]. Table 2 shows the similarities and differences between traditional one-photon PDT and two-photon PDT.

To apply TPA techniques for PDT, a two-photon laser that emits photons at a wavelength that are (i) 2× longer than the PS’s absorbing wavelength, and (ii) falls within the optimal range for tissue penetration (650–800 nm) is employed. Due to their lower energy, the photons involved in TPA have deeper tissue penetration and induce less photo-bleaching of the PS than the shorter wavelength photons [126,127,128]. Also, TPA enables focusing the delivery of these photons (in the form of laser) and thus the activation of PS only at the laser focal point. This gives superior spatial control on the PS activation at the cancer site and prevents photo-induced damage to neighbouring healthy tissues [124]. To date, several attempts have been made to utilise short wavelength-light-activated PSs for PDT cancer treatment using a two-photon light source [125,129,130,131]. For example, a study done by Kobuke and co-researchers (2007) proved that zinc porphyrin derivatives which connect two porphyrins through a bis-acetylene or monoacetylene bond and form a porphyrin dimer through imidazolyl coordination to zinc are able to degrade the HeLa cell membrane under 2 mW light irradiation for 5 min at 780–890 nm [132]. Besides, Anderson et al. (2009) synthesised a series of zinc porphyrin dimers with high singlet oxygen quantum yields. These compounds showed an anticancer and phototoxic effect towards human ovarian adenocarcinoma cell lines at 650–800 nm via TPA activation [133,134,135]. Also, Chao and Gasser (2020) have demonstrated that mice bearing extremely difficult to treat tumours could be eradicated with a Ru(II) polypyridyl complex using two-photon irradiation [136].

However, more mature optical technology for the delivery of two-photon light for clinical PDT is not yet available. On top of that, in the lab, the current achievable two-photon excitation volumes are still relatively small. Thus, this recent advancement of pulsed fibre-optic lasers such as that used as the light delivery method for multiphoton microscopy utilising static infrared beam through in vivo studies especially on the skin of human volunteers may assist in addressing this need [137].

To date, several groups designed a series of transitional metal complexes including ruthenium (Ru) complexes and Re complexes in TPA studies. The published complexes showed a significant phototoxic effect on the tested cell lines. The group of Qiu et al. (2019) reported four Ru(II) polypyridyl complexes having a morpholine moiety and increasingly bipyridine ligand, which possess marked phototoxic effect towards A549 cells. The Ru(II) complex with the most bipyridine ligand showed a better phototoxic effect (0.4 µM) and efficiency (274 GM) in TPA among other synthesised Ru(II) complexes towards A549 cells [138]. Gassser and co-workers (2017) showed that [Ru(phen)_2_dppz]^2+^ derivatives (phen = 1,10-phenanthroline, dppz = dipyrido[3,2-a:2′,3′-c]phenazine) with functional groups on the dppz ligand [dppz-7,8-(OMe)^2^] had a large value of TPA cross-section (245 GM) and better phototoxic effect with TPA on 3D multicellular spheroid in vitro (a better cell model to assess TPA studies)(9.5 µM) as compared to traditional one-photon PDT (32.5 µM). After light irradiation, accumulation of this synthesised Ru(II) complex in the nucleus signifies that the oxidative damage that occurred is severe enough to cause cell necrosis [139,140].

As far as we know, there is no Re(I) complex used in PDT via TPA excitation. However, Lokawicz and co-workers (1999) have described the Re(I) tricarbonyl complex with bipyridine ligand excitable with TPA since the 1990s [141]. A more recent study done by Ferri et al. (2010) proved that the photostable dinuclear Re(I) complex containing a carboxyl group on the diazine ligand conjugated with a peptide nucleic acid conjugate can be excited at 750 nm via TPA (power: 80 mW) [142]. Consequently, it would be worth investigating the Re(I) complex as a potential PS candidate for TPA application in PDT.

### 6.2. Anticancer Combinatorial Therapy

The combination of PDT with chemotherapy has received increased attention as a novel promising approach for cancer treatment. Presently, cisplatin, gemcitabine or doxorubicin are the common chemotherapeutic agents used to treat most cancer which includes pancreatic, breast and bladder cancer. Nevertheless, chemotherapy can cause toxicity in normal cells, lowering the bioavailability of the drug making it less available to the cells and thus, causes poor treatment efficacy or even the possibility of developing multidrug resistance (MDR) [143,144,145,146,147]. In view of this, combined therapy has advantages over a single therapeutic agent, notably, by reducing the possibility of multidrug resistance, enhancing cancer therapeutic efficacy, lowering drug doses needed for treatment and subsequently, and lowering the side effects of the drugs [148]. The synergistic effects of PDT and chemotherapeutic agents allow the selective killing of cancer cells as PDT photosensitisers possess the benefit to selectively kill cancer cells with the generation of ROS following light activation. As PDT derived ROS-based intracellular damage is severe and multi-targeted, DNA damage and repair are expected. In such instances, the efficacies of antimetabolites such as gemcitabine may very much possibly be amplified, as more gemcitabine will be adopted into the DNA repair machinery to induce masked chain termination and cell death.

A recent study by Amin et al. 2019 proved that with a low dose of laser (5 J cm^−2^), higher synergism between doxorubicin and zinc phthalocyanine (ZnPc) were observed when compared to the high dose of laser (20 J cm^−2^) on human melanoma cells (SK-MEL-3). A significant synergy effect was seen with increased sensitivity towards the tested cell line and hence, a lower dose of doxorubicin is required. This results in a lower risk of serious side effects as the resistance of SK-MEL-3 cells are reduced significantly. Besides, it was shown that combination therapy of ZnPc and doxorubicin trigger some anticancer biological processes including increased cell apoptosis, decreased cell migration ability, and autophagy activation [149]. Transition metal complexes can be synergistically paired with current chemotherapeutic agents for anticancer combination therapy. For instance, NAMI-A (imidazolium-*trans*-tetrachloro(dimethylsulfoxide) imidazoleruthenium(III)) was combined with gemcitabine to treat non-small cell lung cancer patients (phase I/II clinical trial). Although patients were found to tolerate the combination treatment moderately, additional studies with larger groups of patients are necessary to draw a decisive conclusion on the anticancer effect of the Re complexes [150].

Re(I) complexes have the potential to be developed in combination therapy. Nevertheless, to our best knowledge, none have tried utilising the potential Re(I) complex in combination therapy with other chemotherapeutic drugs. Hence, more studies can be done to determine the combined therapy effectiveness between luminescent Re(I) complex and currently approved drugs.

### 6.3. Advancement in Nanoparticles with Photosensitisers Utilising Drug Combination Study

Nowadays, the introduction of nanomedicine opens a new avenue towards cancer therapeutic approaches. Nanomedicine brings a solution towards the issue shown by conventional chemotherapeutic agents such as low bioavailability, low lipophilicity, and non-targeted chemotherapy which kills both cancer and healthy cells [151]. Nanoparticle-mediated therapy is an advanced therapeutic approach, which can improve drug tumour selectivity, bioavailability and lower the possibility of drug-causing side effects due to the killing effect towards healthy cells previously [152]. Nanoparticles are able to target tumours selectively, which enables the drugs to accumulate in cancer cells either through active or passive targeting, without being eliminated by the body [152]. Thus, this increases drug concentration in the tumour and substantially enhances the drug cytotoxic effect, which results in lower drug doses required for cancer therapies. As such, the possibility of overcoming multiple drug resistance also increases [152,153]. For instance, paclitaxel, an antimitotic drug, has been approved to be formulated in different types of nano-formulation, which includes albumin-based nanoparticles (Abraxane^®^), liposomes (Lipusu^®^), polymeric micelles (Nanoxel^®^, Genexol^®^, Paclical^®^) to treat pancreatic, breast and non-small-cell lung cancers [154,155]. Nano-drug-delivery systems may improve the efficacy and reduce the adverse effects of anticancer combinatorial therapy through selective and simultaneous delivery of two or more therapeutic drugs to the tumour at a synergistic ratio [151].

In relation to PDT, co-delivery of PS-chemotherapeutic drugs combination has been recently attempted. For instance, Wang and co-workers (2019) have synthesised doxorubicin-loaded PEGylated BODIPY nanoparticles (PEG: polyethylene glycol; BODIPY: distyryl boron dipyrromethene) in which doxorubicin is a chemotherapeutic agent while BODIPY act as photosensitive agents. This synthesised formulation demonstrated synergistic effect and better anticancer activity towards HeLa cells (IC_50_ of 10 nM) when compared to BODIPY alone (IC_50_ of 25 nM) under LED light irradiation for 30 min (20 mW cm^−2^) [148]. Besides, Gaio et al. (2019) have also reported a strong synergistic effect between docetaxel (drug) and Chlorin e6 (Ce-6, photosensitiser) towards HeLa cells. Both mentioned drugs are encapsulated in keratin-based nanoparticles and tested with HeLa parental and resistant cell lines. It demonstrated improvement in the killing effect towards both cell lines after encapsulating docetaxel and Ce-6 into keratin nanoparticles (after formulation of both agents: (IC_50_ of HeLa parental: 0.70 µM; IC_50_ of HeLa resistant: 2.57 µM); docetaxel nanoparticles only: (IC_50_ of HeLa parental: 0.72 µM; IC_50_ of HeLa resistant: 4.16 µM); Ce-6 nanoparticles only: (IC_50_ of HeLa parental: 5.87 µM; IC_50_ of HeLa resistant: 6.27 µM)) [156]. Therefore, utilisation the advancement of combination therapy and nanomedicine to develop a better anticancer agent can be further studied to improve treatment outcomes and relieve patient suffering from the side effects of therapies.

The aforementioned examples have demonstrated enhancements in anticancer combinatorial effects following the co-integration of PS and chemotherapeutic drugs within the nano-drug-carriers. In light of this, co-integration of Re(I) complexes along with other anticancer drugs at a synergistic ratio may be a feasible approach to attempt, for the enhancement of its anticancer efficacy.

### 6.4. Photothermal Therapy

Photothermal therapy (PTT) is another effective, non-invasive way to treat cancers whereby it utilises a photosensitive agent to turn photonic energy into heat energy and induce thermal ablation upon light irradiation [157]. It works by elevating the ground-state electrons to an excited state through photonic energy absorption and then the kinetic energy release and overheat the cells, which results in cell and tissue damage [158]. In PTT, hyperthermia (with an intracellular temperature of cells of around 50 °C) can cause the cell membrane to lyse and thus, cause necrotic or apoptotic cell death [159,160,161]. In comparison with PDT, PTT does not require oxygen to kill the cancer cells but it generates the cytotoxic effect through an increase in the temperature.

Saw and co-researchers (2017) have reported that citric acid-coated confeito-like gold nanoparticles with 30 nm has the potential to become a PTT agent. The synthesised nanoparticles with 30 nm show the best in vitro PTT efficacy on MDA-MB-231 cells with laser irradiation at 532 nm for one minute as compared to the other size of nanoparticles (60, 80, 100 nm). It also displayed higher localisation at endoplasmic reticulum which further enhances endocytosis towards the cancerous cells [162]. For instance, Zhao et al. (2018) have published one of the transition metal complexes, ruthenium nanoparticles with good biocompatibility and was easily metabolised to the PTT field. With 10 min light irradiation at 808 nm on A549 cells, the ruthenium nanoparticles induce significant cell death, cell ablation and destruction in in vitro and in vivo experiments [163]. Thus, it is possible for the Re(I) complex to be tested on cancer cells through PTT as it possesses similar properties to ruthenium since they both are neighbours in the periodic table. PTT might be another option for the Re(I) complex to exhibit its unique characteristics to kill the cancer cells.

## 7. Conclusions

The development of Re(I) tricarbonyl complexes has unlocked a new avenue for the treatment of cancer via photodynamic therapy to improve the overall survival rate of cancer patients. To achieve this, researchers and clinicians have to improvise their understandings of tumour immunology, novel complexes, and optimise the timing of photodynamic therapy. We believed that these novel complexes represent a promising alternative in all types of cancer, thus progressing from bench to bedside at a swift pace. Re(I) complexes as photosensitisers in photodynamic therapy will bring a bright future ahead for patients suffering from this aggressive and challenging killer.

## Figures and Tables

**Table 1:** Application of clinically approved photosensitisers for treatment of cancer.

Photosensitisers	Application	References
NPe6 (Talaporfin sodium)	Non-small cell lung carcinoma	[37]
Motexafin lutetium	Prostate cancer	[38]
Temoporfin	Head, neck, prostate and pancreatic cancers	[39,40,41]
Porfimer sodium	Obstructive oesophageal, lung, bladder and cervical cancers	[28,42,43]
2-(1-Hexyloxyethyl)-2-devinyl pyropheophorbide-a	Head, lung and neck cancers, basal cell carcinoma	[44,45,46]
Hexaminolevulinate	Bladder cancer	[47]
Methyl aminolevulinate	Basal cell carcinoma	[40,48,49]
Aluminium phthalocyanine tetrasulfonate	Lung, breast, skin and stomach cancers	[50]
Padeliporfin	Early-stage of prostate cancer	[51]
Verteporfin	Basal cell carcinoma	[52,53]

**Table 2:** Comparison between traditional one-photon photodynamic therapy and two-photon photodynamic therapy.

**Photodynamic Therapy**			
**One-Photon Photodynamic Therapy**		**Two-Photon Photodynamic Therapy**	
**Similarities**			
The general mechanism is the same, with the presence of light and oxygen, the photosensitisers are excited to its excited triplet state which leads to the production of reactive oxygen species (ROS) and thus, causing cell death.			
**Differences**			
One-photon photosensitiser is used	**Photosensitisers**		Two-photon photosensitiser is used
600–800 nm	**Ideal range of wavelength (nm) of the photosensitisers**		Wide range, not fixed, can go as low as 300 nm
A laser within the UV-visible range	**Activation of photosensitisers**		Two low energy photons of near-infrared region of light absorbed simultaneously
Less	**Precision of cancer treatment**		Higher
Shallower	**Depth of tissue penetration**		Deeper
-	**Determination of ability of photosensitiser to absorb 2 photons simultaneously**		Quantified by two-photon cross-sections, δ, which is expressed in Goeppert-Mayer (GM), best to > 50 GM
